# The novel long noncoding RNA CRART16 confers cetuximab resistance in colorectal cancer cells by enhancing ERBB3 expression via miR-371a-5p

**DOI:** 10.1186/s12935-020-1155-9

**Published:** 2020-03-04

**Authors:** Xiaoqian Zhang, Long Wen, Shanwen Chen, Junling Zhang, Yongchen Ma, Jianwen Hu, Taohua Yue, Jingui Wang, Jing Zhu, Dingfang Bu, Xin Wang

**Affiliations:** 1 Department of General Surgery, Peking University First Hospital, Beijing, 100034 People’s Republic of China; 2 Department of Endoscopic Center, Peking University First Hospital, Beijing, 100034 People’s Republic of China; 3 Central Laboratory, Peking University First Hospital, Beijing, 100034 People’s Republic of China

**Keywords:** Long noncoding RNA, CRART 16, Cetuximab, Resistance, miR-371a-5p, ERBB3

## Abstract

**Background:**

Long noncoding RNAs (lncRNAs) have been shown to participate in multiple biological processes and confer drug resistance. However, it remains unclear whether lncRNAs are involved in conferring cetuximab resistance in colorectal cancer (CRC) cells.

**Methods:**

Cell Counting Kit-8 (CCK-8) assays were performed to assess the sensitivity of CRC cell lines to cetuximab treatment. We incubated Caco-2 cells, which are partially responsive to cetuximab, with increasing concentrations of cetuximab for approximately 6 months to generate Caco-2 cetuximab-resistant (Caco-2 CR) cells. Microarray analysis comparing Caco-2 CR with Caco-2 cells was used to identify lncRNAs that were potentially related to cetuximab resistance. Caco-2 cells were stably transduced with cetuximab resistance-associated RNA transcript 16 (CRART16) or an empty vector using lentiviral infection; the cells were designated Caco-2-CRART16 and Caco-2-NC, respectively, and were analyzed with RNA sequencing (RNA-seq). Quantitative real-time PCR (qRT-PCR) was performed to investigate RNA expression. Flow cytometry and TUNEL assays were used to assess apoptosis levels induced by cetuximab. The cell cycle, stemness biomarkers and membrane proteins of CRC cells were assessed via flow cytometry. RNA fluorescence in situ hybridization (FISH) was used to examine CRART16 localization and expression. Bioinformatics analysis was performed to predict the potential mechanism of CRART16, which was further validated by a dual-luciferase reporter assay. Differences in measurement data were compared using Student’s t test, one-way ANOVA followed by Dunnett’s test and two-way ANOVA.

**Results:**

The novel lncRNA CRART16 was upregulated in Caco-2 CR cells. CRART16 overexpression reversed the effects of cetuximab on cell viability and reduced cetuximab-induced apoptosis. Meanwhile, CRART16 overexpression led to increases in the proportion of CD44^+^/CD133^+^ cells. In addition, CRART16 acts as a competing endogenous RNA (ceRNA) for miR-371a-5p to regulate V-Erb-B2 Erythroblastic Leukemia Viral Oncogene Homolog 3 (ERBB3) expression. MiR-371a-5p mimics counteracted the cetuximab resistance induced by CRART16 overexpression. Kyoto Encyclopedia of Genes and Genomes (KEGG) pathway analysis revealed that after CRART16 was overexpressed, the resulting differentially expressed mRNAs were mainly enriched in the MAPK signaling pathway.

**Conclusions:**

CRART16 overexpression may contribute to cetuximab resistance through the miR-371a-5p/ERBB3/MAPK pathway. Additionally, CRART16 contributes to the acquisition of stemness properties.

## Background

Colorectal cancer (CRC) is the third most commonly diagnosed cancer (after lung cancer and breast cancer), with 1.8 million newly diagnosed cases annually, and is the second leading cause of cancer-related death according to GLOBOCAN 2018 [1]. In the past two decades, with the use of chemotherapeutic drugs and the development of treatments, the overall survival (OS) of metastatic colorectal cancer (mCRC) patients has been prolonged to approximately 2 years [2]. The application of targeted agents, such as cetuximab and bevacizumab, further improved the OS of mCRC patients to approximately 30 months [3, 4]. Cetuximab, an immunoglobulin G1 (IgG1) monoclonal antibody against epidermal growth factor receptor (EGFR), competitively binds to the extracellular domain of EGFR, thereby attenuating ligand-induced EGFR tyrosine kinase activity [5] and blocking downstream RAS–RAF–MAPK, PI3K–PTEN–AKT and JAK–STAT3 signaling [5, 6]. However, approximately 80% of mCRC patients who harbor KRAS, NRAS, BRAF and PIK3CA gene mutations do not benefit from cetuximab treatment [7, 8], and almost all patients who are sensitive to cetuximab will progress within 3–12 months [9]. More recently, attention has been given to the mechanism underlying the development of acquired resistance to cetuximab, and it remains a promising approach for seeking novel therapeutic targets for late-stage CRC.

Long noncoding RNAs (lncRNAs), which are longer than 200 nucleotides (nt) and do not have protein-coding potential [10], have been implicated in various biological processes [11], associated with different cancer types [12] and involved in drug resistance [13]. Moreover, the functions of lncRNAs are summarized into four archetypes: signals, decoys, guides, and scaffolds [14]. The specific functions of lncRNAs are related to their subcellular localization [15]. In-cis-accumulated lncRNAs can act in *cis* or in *trans* once they are transcribed. The lncRNAs those localize in the nucleoplasm in trans and accumulate to specific nuclear bodies can act in *trans*. Moreover, cytoplasmic lncRNAs can interfere with protein posttranslational modifications, regulate gene expression by binding microRNAs (miRNAs) and proteins, affect mRNA translation, etc. LncRNAs participate in gene regulation by serving as miRNA sponges, a molecular mechanism known as competing endogenous RNAs (ceRNAs) [16]. The lncRNA CRNDE was shown to promote CRC cell proliferation and chemoresistance by regulating miR-181a-5p in vitro [17]. The lncRNA SNHG6 was reported to promote CRC cell growth, migration, and invasion both in vitro and in vivo by interacting with miR-26a, miR-26b, and miR-214 and regulating their common target EZH2 [18].

In this study, we found that ENST00000564193.1 was upregulated after prolonged cetuximab stimulation in Caco-2 cells. Based on this finding, we named this novel transcript lncRNA cetuximab resistance-associated RNA transcript 16 (CRART16). In addition, the overexpression of CRART16 induced cetuximab resistance by downregulating miR-371a-5p, which negatively regulates the expression of V-Erb-B2 Erythroblastic Leukemia Viral Oncogene Homolog 3 (ERBB3).

## Materials and methods

### Cell lines and cell culture

The human CRC cell lines HCT116, HT29, HCT8, SW620 and Caco-2 were purchased from the Cancer Institute of the Chinese Academy of Medical Science. HT29, HCT8, SW620 and Caco-2 cells were cultured in Dulbecco’s modified Eagle’s medium–high glucose (DMEM, Thermo Fisher Scientific, MA, USA), while HCT116 cells were grown in McCoy’s 5A Medium (Thermo Fisher Scientific, MA, USA). DMEM and McCoy’s 5A were both supplemented with 10% fetal bovine serum (FBS, Thermo Fisher Scientific, MA, USA) and 1% penicillin–streptomycin (Thermo Fisher Scientific, MA, USA). All cells were cultured in a humidified incubator with 5% carbon dioxide (CO_2_) and 95% air at 37 °C. Caco-2 cetuximab-resistant (Caco-2 CR) cells was generated by exposing cells to increasing concentrations of cetuximab at a constant concentration of 200 μg/ml over 6 months. The residual colonies were designated Caco-2 CR.

### Microarray analysis

Total RNA was extracted from Caco-2 and Caco-2 CR cells using TRIzol reagent (Invitrogen, Carlsbad, CA, USA). The RNA purity and concentration were determined with OD260/280 readings using a spectrophotometer. Complementary DNA (cDNA) was labeled with Cy3-dCTP using Eberwine’s linear RNA amplification method and an enzymatic reaction. Then, amplified complementary RNA (cRNA) was transcribed from double-stranded cDNA (dsDNA). After reverse transcription, the Klenow enzyme labeling strategy was adopted using CbcScript II reverse transcriptase. Subsequently, arrays loaded with labeled cDNA were hybridized in an Agilent hybridization oven overnight. Array data were analyzed by GeneSpring software V13.0 (Agilent). After data summarization, normalization and quality control, gene expression data were log2-transformed and median-centered by genes using the adjust data function of CLUSTER 3.0 software.

### RNA isolation, library construction and sequencing

Total RNA was extracted using TRIzol reagent following the manufacturer’s instructions. RNA degradation and contamination were monitored on 1% agarose gels. The purity of RNA was verified using a NanoPhotometer spectrophotometer (IMPLEN, CA, USA). The concentration of RNA was measured using a Qubit^®^ RNA Assay Kit with a Qubit^®^ 2.0 Fluorometer (Life Technologies, CA, USA). RNA integrity was assessed using an RNA Nano 6000 Assay Kit and an Agilent Bioanalyzer 2100 system (Agilent Technologies, CA, USA). A total amount of 3 µg RNA per sample was used as the input material for RNA sample preparation and for small RNA libraries. Sequencing libraries were generated using an NEBNext^®^ Ultra™ RNA Library Prep Kit for Illumina^®^ (NEB, USA) and an NEBNext^®^ Multiplex Small RNA Library Prep Set for Illumina^®^ (NEB, USA.) following the manufacturer’s recommendations, and index codes were added to attribute specific sequences to each sample. The clustering of index-coded samples was performed on a cBot Cluster Generation System using a TruSeq PE Cluster Kit v3-cBot-HS (Illumina) according to the manufacturer’s instructions. After cluster generation, libraries were sequenced on an Illumina platform, and 125 bp/150 bp paired-end reads were generated. Differential expression analysis of two groups was performed using the DESeq2 R package (1.16.1).

### Lentivirus transduction

The plasmid pCDH-CMV-MCS-EF1-GFP+Puro, which contained the full-length CRART16 cDNA, and an empty vector were purchased from Mailgene Biosciences Co., Ltd. (Beijing, China). 293T cells were transfected with a psPAX2-pMD2.G lentiviral vector packaging system to produce lentivirus. The viral supernatant was collected at 24 h and 48 h after transfection and filtered through a 0.45-μm PVDF filter. The viral supernatant was mixed with PEG-8000 (Solarbio, Beijing, China) and incubated overnight at 4 °C. Lentiviruses were harvested by centrifugation at 8000 rpm for 30 min and then resuspended in complete medium. Caco-2 cells were infected with lentiviruses in the presence of 5 μg/ml polybrene (Sigma-Aldrich, MO, USA). Twenty-four hours after infection, the infectious medium containing lentiviruses was replaced with complete medium. After confirming GFP expression in Caco-2 cells, puromycin selection was performed at 6.5 μg/ml for at least 2 weeks. After stable transfection was completed, Caco-2 cells overexpressing CRART16 and negative control cells were named Caco-2-CRART16 cells and Caco-2-NC cells, respectively. The expression of CRART16 in Caco-2-CRART16 cells and Caco-2-NC cells was measured by quantitative real-time PCR (qRT-PCR).

### RNA extraction and quantitative real-time PCR analyses

Total RNA was extracted from CRC cells using TRIzol reagent (Invitrogen, Carlsbad, CA, USA) according to the manufacturer’s instructions. Four micrograms of total RNA were reverse transcribed into cDNA using a RevertAid RT Reverse Transcription Kit (Thermo Fisher Scientific, MA, USA) and TransScript miRNA First-Strand cDNA Synthesis SuperMix (Transgen Biotech, Beijing, China). qRT-PCR was performed on an Applied Biosystems 7500 Real-Time PCR System (Applied Biosystems) using PowerUp™ SYBR™ Green Master Mix (Thermo Fisher Scientific, MA, USA). The relative expression of lncRNAs and mRNAs was normalized to that of GAPDH. U6 small nuclear RNA (snRNA) was used as an internal control for miRNA in each sample. The relative concentrations of RNAs were calculated using the comparative cycle threshold (CT) (2^−△△CT^) method. Primer sequences are provided in Table 1.

**Table 1:** Primer sequences used for qRT-PCR

Gene	Sequence of the primers
lncRNA CRART16, forward primer	5′-TGATAGTGAGGCCTCCTGCAA-3′
lncRNA CRART16, reverse primer	5′-CTGGAGTTCTGCAGGTTCCTTT-3′
miR-371a-5p, forward primer	5′-ACTCAAACTGTGGGGGCACT-3′
U6, forward primer	5′-GCAAGGATGACACGCAAATTC-3′
ERBB3, forward primer	5′-CTCCGAGGTGGGCAACTCT-3′
ERBB3, reverse primer	5′-TGTACAGTGTCTGGTATTGGTTCTCA-3′
ATP8B1, forward primer	5′-GAGAACCGGGAGCCATTCA-3′
ATP8B1, reverse primer	5′-AAGTGAGGTTGTTCGTGGTACTTG-3′
KAT6A, forward primer	5′-TGTTGTGATCCGCCACTCA-3′
KAT6A, reverse primer	5′-TCCTTTTTTCCTAGGTCGACATATTT-3′
FKTN, forward primer	5′-AGGAAGCCGAATTGGATTTGA-3′
FKTN, reverse primer	5′-CACTGGTACATTTTGGTTGGATGT-3′
UCHL5, forward primer	5′-TCCCGACTTGACACGATATTTTT-3′
UCHL5, reverse primer	5′-TGGTGGGTACAGTTCAGTAACACA-3′
NUF2, forward primer	5′-GCTGATGGTAAAAACCTCACCAA-3′
NUF2, reverse primer	5′-GCTCTCATGTAGATCATGTGCAAGA-3′
RPS6KA3, forward primer	5′-ACCTATGGGAGAGGAGGAGATTAAC-3′
RPS6KA3, reverse primer	5′-CCTTTACATGATGTGTGATTGCAAT-3′

### Cell viability assay

Exponentially growing cells were seeded in 96-well plates at a density of 3000 cells in 100 μl complete medium. Twenty-four hours after cell plating, cells were incubated with graded concentrations (0–200 μg/ml) of cetuximab (Merck KGaA, Darmstadt, Germany) for 48 h. Cell Counting Kit-8 (CCK-8; Bimake, Shanghai, China) reagent was added to the 96-well plates and incubated for 1–3 h at 37 °C. The absorbance was measured at 450 nm and recorded. Each concentration had six replicates, and the experiment was repeated at least three times.

### Flow cytometry analysis of apoptosis and the cell cycle

Cells were plated in T25 flasks at a density of 1.5 × 10^5^ cells in 4 ml complete medium. Twenty-four hours after cell plating, cells were incubated with or without 200 μg/ml cetuximab for 48 h. Apoptotic cells were measured by flow cytometry (BD Biosciences, NJ, USA) after staining with APC Annexin V and 7-amino-actinomycin D (7-AAD, BD Biosciences, NJ, USA) following the manufacturer’s instructions. In the analysis of the apoptotic status, APC Annexin V^−^/7-AAD^−^ denotes live cells; APC Annexin V^+^/7-AAD^−^ denotes early apoptotic cells; APC Annexin V^−^/7-AAD^+^ denotes necrotic cells; and APC Annexin V^+^/7-AAD^+^ denotes late apoptotic cells. For cell cycle analysis, cells were harvested and incubated with 75% ethanol overnight at 4 °C and stained with propidium iodide (PI)/RNase Staining Buffer (BD Biosciences, NJ, USA) following the manufacturer’s instructions. Each experiment was repeated three times.

### Flow cytometry analysis of membrane proteins

CD44 and CD133 were measured by a FACSCalibur flow cytometer (BD Biosciences, NJ, USA) to evaluate the percentage of cancer stem cell (CSC)-like cells. EGFR, ERBB3 and c-MET were measured by Gallios (Beckman). Single-cell suspensions were prepared and incubated with Human TruStain FcX™ (Fc Receptor Blocking Solution, BioLegend, CA, USA) at 5 μl per 10^6^ cells in 100 μl PBS at room temperature for 10 min. Anti-CD44-APC (BioLegend, CA, USA) and anti-CD133-PE (BioLegend, CA, USA) or anti-human EGFR-PerCP/Cyanine5.5 (BioLegend, CA, USA), anti-human erbB3/HER-3-PE (BioLegend, CA, USA) and anti-human c-MET-AF647 (BD Biosciences, NJ, USA) were added at a constant concentration of 5 μl antibody/10^6^ cells/100 μl PBS and incubated in the dark at room temperature for 15 min. Negative controls were stained with corresponding isotype control products. All experiments were repeated three times. In this study, the fluorescence intensity data were approximately normally distributed, so the arithmetic mean fluorescence intensity (MFI) was used to indicate the expression of membrane proteins.

### TUNEL

A total of 2 × 10^4^ cells was seeded onto coverslips in 24-well plates. Coverslips were fixed with 4% paraformaldehyde for 30 min at room temperature. Endogenous peroxidase was blocked with methanol and 30% H_2_O_2_ at a 50:1 ratio for 30 min at room temperature. Cell apoptosis was assessed by a TUNEL Apoptosis Detection Kit I, POD (Boster, California, USA) as per the manufacturer’s instructions. Then, slides were incubated with diaminobenzene (DAB, Boster, California, USA) followed by counterstaining with hematoxylin (Solarbio, Beijing, China). After dehydration in an ethanol series and clearing with xylene, coverslips were mounted with mounting medium (GSGB Bio, Beijing, China).

### Transient transfection

Transient transfection was performed using Lipofectamine 3000 reagent (Invitrogen, Carlsbad, CA, USA) according to the manufacturer’s protocol. Cells were transfected with double-stranded miR-371a-5p mimics and negative control RNA (miR-NC) (GenePharma, Shanghai, China).

### Fluorescence in situ hybridization (FISH)

Two pairs of primers specific for CRART16 were designed, and their specificity was confirmed by NCBI BLAST (Table 2). Two specific fragments for CRART16 were generated by the PCR amplification of genomic DNA from a normal, healthy person, and these fragments were cloned into a TA cloning vector. Clones were selected for sequencing, and then gene-specific plasmids were linearized. Linearized plasmids were labeled with PCR Fluorescein Labeling Mix (Roche, Basel, Switzerland) by PCR as per the manufacturer’s instructions. Then, the bands of PCR products were detected at the expected positions by agarose gel electrophoresis. In addition, slides were sterilized by immersion in 75% alcohol and exposure to ultraviolet light. CRC cells were seeded on slips in 10-cm dishes and harvested in the logarithmic phase. After incubating at 56 °C for 30–60 min, specimens were fixed with methanol and glacial acetic acid at a 3:1 ratio for 20 min at room temperature. Then, the slides were washed with 2× SSC for 30 min, dehydrated in a gradient ethanol series and incubated at 56 °C. The labeled DNA was dissolved in a hybridization solution composed of 50% deionized formamide, 5× SSC, 5× denhardt, 0.5% SDS, 100 μg/ml salmon sperm DNA and 10% dextran sulfate and was denatured at 73 °C for 5 min. Specimens were hybridized according to the manufacturer’s instructions. Subsequently, the slides were mounted with antifading solution containing DAPI. Signals were detected by confocal laser scanning microscopy.

**Table 2:** Primer sequences for FISH probes

Probe	Primer	Sequence
Probe A	Forward primer	5′-CACACCTTGTGCTTCCATAGAATT-3′
	Reverse primer	5′-CTGGTCTGTGGTGTTTGTTATAGCC-3′
Probe B	Forward primer	5′-GGAACCTGCAGAACTCCAGG-3′
	Reverse primer	5′-CCCAGCACACGTGACTTGATAG-3′

### Dual-luciferase assay

The full-length sequence of CRART16 and the ERBB3 3′ untranslated region (3′-UTR) were subcloned into the pmiR-RB-Report™ vector (Ribobio, Guangzhou, China). The WT vector or the empty vector and miR-371a-5p mimics or NC were cotransfected into 293T cells using Lipofectamine 3000 reagent following the manufacturer’s instructions. Forty-eight hours after transfection, the luciferase activity was assessed using a Dual-luciferase Reporter Assay System (Promega, Madison, Wisconsin, USA).

### Statistical analysis

All statistical analyses were performed using the SPSS 24.0 statistical software package (Chicago, IL). Data are presented as mean ± standard deviation (SD). P values were two-sided and considered significant at a level of 0.05. Differences in measurement data were compared using Student’s t test, one-way ANOVA followed by Dunnett’s test and two-way ANOVA.

## Results

### Establishment of cetuximab-resistant Caco-2 cells

**Fig. 1:**
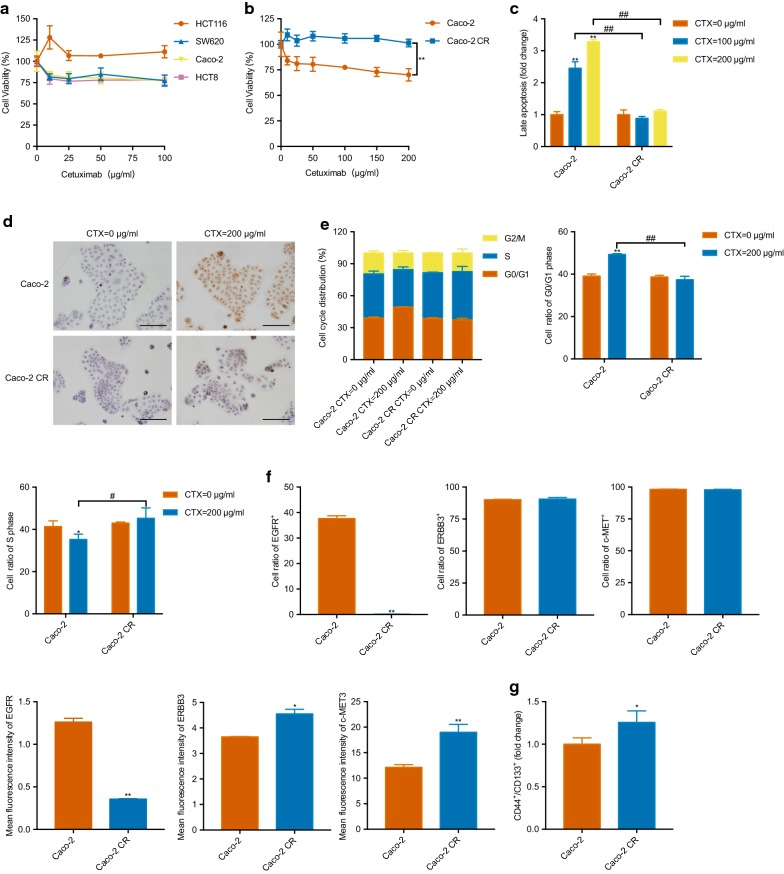
Phenotypic characteristics of Caco-2 and Caco-2 CR cells. **a** The sensitivity of a panel of CRC cell lines (HCT116, SW620, Caco-2 and HCT8) to cetuximab treatment was assessed by CCK8 assays. CRC cells were incubated with graded concentrations (0–200 μg/ml) of cetuximab for 48 h. Six replicates were used for each concentration, and the experiment was repeated at least three times. Data are presented as mean ± SD. **b** Cell viability was assessed by CCK8 assays in Caco-2 and Caco-2 CR cells treated with various concentrations of cetuximab (0–200 μg/ml) for 48 h. Each concentration had six replicates, and the experiment was repeated at least three times. Data are presented as mean ± SD. **P < 0.01 by two-way ANOVA. **c** Flow cytometry was performed in Caco-2 and Caco-2 CR cells with cetuximab treatment (100 μg/ml and 200 μg/ml) for 48 h. All experiments were repeated three times, and data are presented as mean ± SD. **P < 0.01 by Student’s t test. ^##^P < 0.01 by one-way ANOVA followed by Dunnett’s test. **d** Caco-2 and Caco-2 CR cells were treated with or without 200 μg/ml cetuximab (CTX) for 48 h. Apoptosis was detected by a TUNEL assay. Scale bar = 100 μm. **e** The cell cycle was assessed by flow cytometry in Caco-2 and Caco-2 CR cells after 48 h of treatment with cetuximab (200 μg/ml). All experiments were repeated three times, and data are presented as mean ± SD. *^/#^P < 0.05, **^/##^P < 0.01 by Student’s t test. **f** The percentage of EGFR-, ERBB3-, and c-MET-positive cells and the MFI were determined by GALLIOUS flow cytometry in Caco-2 and Caco-2 CR cells. All experiments were repeated three times, and data are presented as mean ± SD. *P < 0.05, **P < 0.01 by Student’s t test. **g** Flow cytometry analysis showed the expression of stemness biomarkers of CRC cells, CD44 and CD133, in Caco-2 and Caco-2 CR cells. All experiments were repeated three times, and data are presented as mean ± SD. *P < 0.05 by Student’s t test

We examined the sensitivity of a panel of CRC cell lines to cetuximab treatment by incubating the cells with various concentrations of cetuximab for 48 h, and then CCK8 assays were performed. Based on dose–response curves, HCT116 cells are intrinsically resistant to cetuximab, while Caco-2, SW620 and HCT8 cells are partially responsive (Fig. 1a). Then, we incubated Caco-2 cells with increasing concentrations of cetuximab for approximately 6 months to generate Caco-2 CR, which were cultured with cetuximab at a constant concentration of 200 μg/ml. Cell viability was assessed by a CCK8 assay (Fig. 1b). Flow cytometry and TUNEL assays were used to assess the extent of apoptosis induced by cetuximab in Caco-2 cells and Caco-2 CR (Fig. 1c and d and Additional file 1: Figure S1a). According to the results, cetuximab mainly induces late apoptosis (APC Annexin V^+^/7-AAD^+^cells in flow cytometry and TUNEL-positive cells) with increasing concentrations of cetuximab. Cell cycle analysis by flow cytometry showed that there were no differences between Caco-2 cells and Caco-2 CR cells, while cetuximab inhibited the proliferation of Caco-2 cells by inducing G_0_/G_1_ cell cycle arrest (Fig. 1e and Additional file 1: Figure S1b). Flow cytometry was performed to evaluate the cell ratio of EGFR^+^, EBBB3^+^ and c-MET^+^ cells and the MFIs of these three membrane proteins. As shown in Fig. 1f and Additional file 1: Figure S1c, the expression of EGFR was blocked by continued stimulation of cetuximab, resulting in a compensatory overexpression of ERBB3 and c-MET. In addition, the percentage of CD44^+^/CD133^+^ cells, which were considered CSC-like cells, was evaluated by flow cytometry. The results indicated that cells with stemness properties were more populated over long-term treatment by cetuximab (Fig. 1g and Additional file 1: Figure S1d).

### Identification of differentially expressed lncRNAs involved in cetuximab resistance

**Fig. 2:**
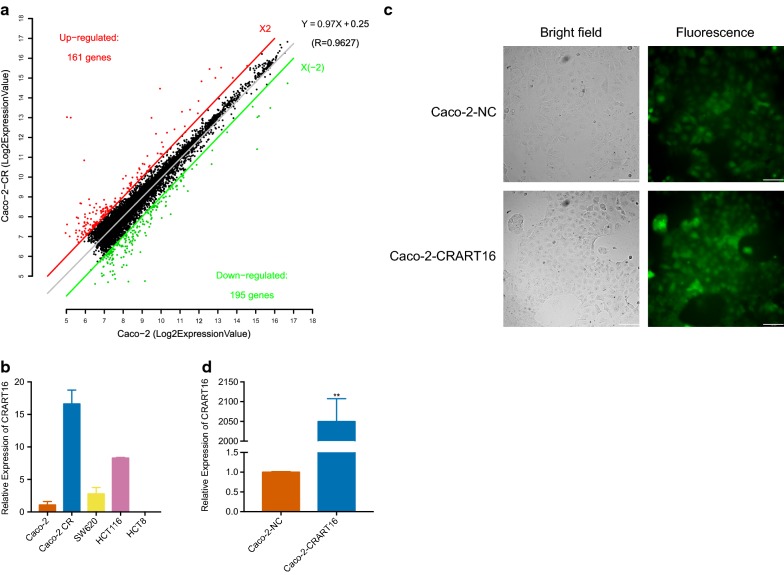
CRART16 is upregulated in Caco-2 CR. **a** Scatter plots show the difference in the expression of lncRNAs between Caco-2 and Caco-2 CR cells. Red dots above the red line indicate significantly upregulated lncRNAs, whereas green dots below the green line indicate significantly downregulated lncRNAs. **b** The expression of the CRART16 was detected in a panel of CRC cell lines (Caco-2, Caco-2 CR, HCT116, SW620 and HCT8) by qRT-PCR. GAPDH was used as an internal control. All experiments were repeated three times, and data are presented as mean ± SD. **c** The transfection efficiency and morphology of Caco-2-CRART16 and Caco-2-NC cells were viewed by bright field and fluorescence microscopy. Scale bar = 100 μm. **d** Transfection efficiency of the lncRNA CRART16 was detected in Caco-2-CRART16 and Caco-2-NC cells by qRT-PCR. GAPDH was used as an internal control. All experiments were repeated three times, and data are presented as mean ± SD. **P < 0.01 by Student’s t test

A lncRNA microarray comparing Caco-2 CR with Caco-2 cells was used to investigate the potential molecular mechanisms of cetuximab resistance. To detect differentially expressed lncRNAs, we used threshold fold change values of ≥ 2 and ≤ −2. As shown in the scatter plots (Fig. 2a), the two groups were compared, and a total of 356 transcripts were differentially expressed, which included 161 upregulated lncRNAs and 195 downregulated lncRNAs.

### Establishment of cell lines with stable CRART16 overexpression

To validate the results of the lncRNA microarray, the expression levels of the top 10 upregulated genes were analyzed by qRT-PCR in CRC cell lines. Our results showed that the novel lncRNA ENST00000564193.1 was the most differentially expressed gene, which was increased by 13.31-fold in Caco-2-CR compared with Caco-2 cells (Fig. 2b). ENST00000564193.1 is 744 bp long, is located on chromosome 16, contains 2 introns and 3 exons, and is designated CRART16 based on the HUGO Gene Nomenclature Committee (HGNC) Guidelines [19]. The expression level of CRART16 in other CRC cell lines was also assessed by qRT-PCR. Our data revealed that CRART16 expression was upregulated not only in Caco-2-CR cells with acquired cetuximab resistance but also in HCT116 cells with primary cetuximab resistance (Fig. 2b). Then, we stably transduced Caco-2 cells with CRART16 using lentiviral infection, and the cells were designated Caco-2-CRART16. Caco-2 cells that were transfected with an empty vector were designated Caco-2-NC. The transfection efficiency was determined by fluorescence microscopy and qRT-PCR (Fig. 2c, d).

### CRART16 promotes cetuximab resistance in CRC cells and contributes to the acquisition of stemness properties

**Fig. 3:**
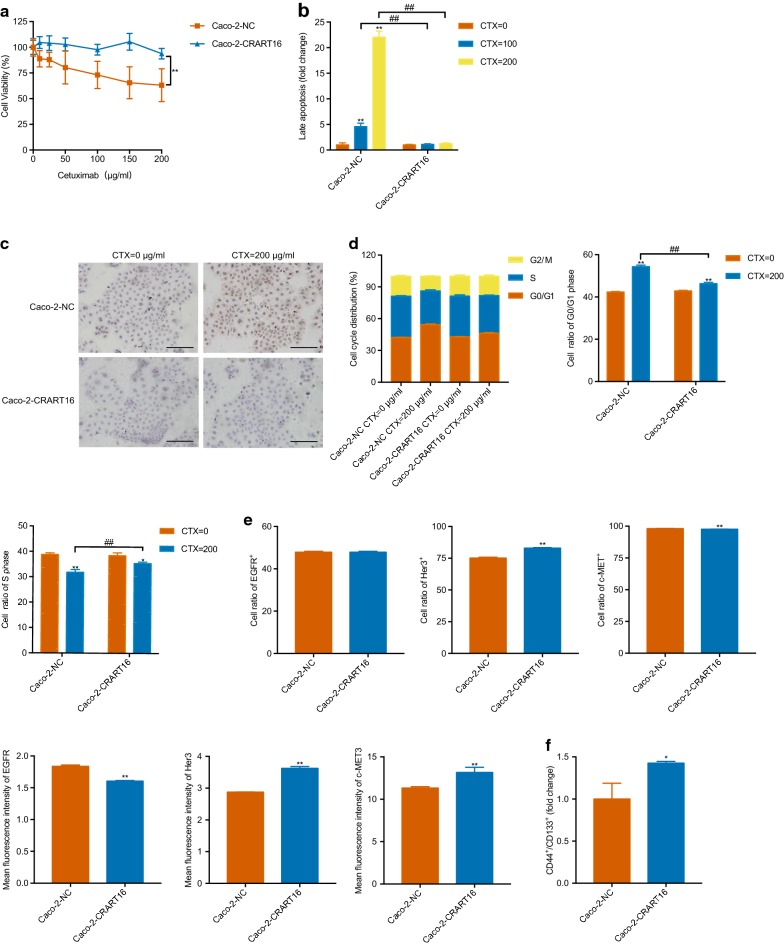
CRART16 promotes cetuximab resistance and contributes to the acquisition of stemness properties of CRC cells. **a** CCK8 assay was performed to assess the cell viability of Caco-2-CRART16 and Caco-2-NC cells treated with graded concentrations of cetuximab (0–200 μg/ml) for 48 h. Each concentration had six replicates, and the experiment was repeated at least three times. Data are presented as mean ± SD. **P < 0.01 by two-way ANOVA. **b** Flow cytometry was performed in Caco-2-CRART16 and Caco-2-NC cells with cetuximab treatment (100 μg/ml and 200 μg/ml) for 48 h. All experiments were repeated three times, and data are presented as mean ± SD. **^/##^P < 0.01 by Student’s t test. **c** Caco-2-CRART16 and Caco-2-NC cells were treated with 200 μg/ml cetuximab for 48 h. Apoptosis was detected by a TUNEL assay. Scale bar = 100 μm. **d** The cell cycle was assessed by flow cytometry in Caco-2-CRART16 and Caco-2-NC cells after 48 h of treatment with cetuximab (200 μg/ml). All experiments were repeated three times, and data are presented as mean ± SD. *P < 0.05, **^/##^P < 0.01 by Student’s t test. **e** The percentage of EGFR-, ERBB3-, and c-MET-positive cells and the MFI were determined by a GALLIOUS flow cytometer in Caco-2-CRART16 and Caco-2-NC cells. All experiments were repeated three times, and data are presented as mean ± SD. **P < 0.01 by Student’s t test. **f** Flow cytometry analysis showed the expression of stemness biomarkers in CRC cells, CD44 and CD133, in Caco-2-CRART16 and Caco-2-NC cells. All experiments were repeated three times, and data are presented as mean ± SD. *P < 0.05 by Student’s t test

To determine whether CRART16 was involved in cetuximab resistance, a CCK8 assay was used to assess cell viability, and the results showed that Caco-2-CRART16 cells were more resistant to cetuximab than Caco-2-NC cells (Fig. 3a). After treatment with cetuximab for 48 h, compared with Caco-2-CRART16 cells, Caco-2-NC cells had an increased proportion of late apoptotic cells (Fig. 3b and Additional file 2: Figure S2a). In addition, the TUNEL assay results were highly consistent with the flow cytometry results (Fig. 3c). Furthermore, the overexpression of CRART16 did not cause significant changes in cell cycle distribution but could inhibit the G_0_/G_1_ phase cell cycle arrest caused by cetuximab (Fig. 3d and Additional file 2: Figure S2b). The MFIs of ERBB3 and MET were higher in Caco-2- CRART16 cells than in Caco-2-NC cells, whereas the MFI of EGFR was lower (Fig. 3e and Additional file 2: Figure S2c). Notably, CRART16 overexpression led to an increase in the proportion of CD44^+^/CD133^+^ cells (Fig. 3f and Additional file 2: Figure S2d). Collectively, these data demonstrated that CRART16 confers cetuximab resistance to CRC cells and contributes to the acquisition of stemness properties.

### CRART16 functions as a miR-371a-5p sponge

**Fig. 4:**
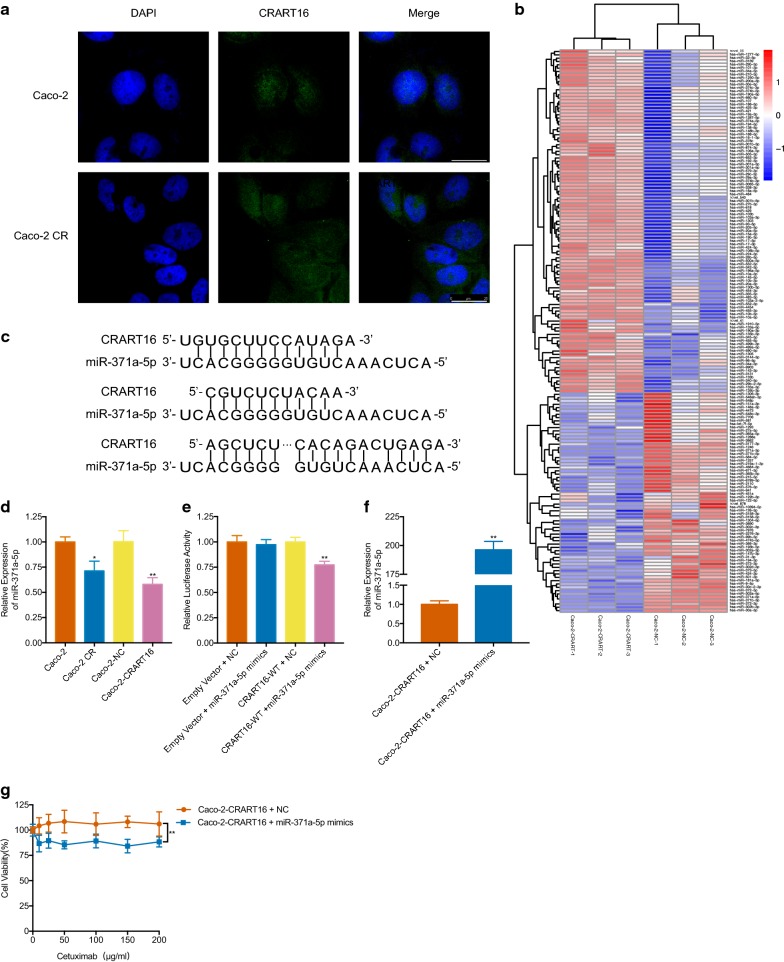
CRAT16 functions as a miR-371a-5p sponge. **a** FISH analysis of CRART16 in Caco-2 and Caco-2 CR cells (nuclei were stained with DAPI). Scale bar = 25 μm. **b** MiRNA heatmap shows the top differentially expressed miRNAs in Caco-2-CRART16 cells versus Caco-2-NC cells. **c** Schematic diagram of the predicted binding sites between lncRNA CRAT16 and miR-371a-5p. **d** The expression of miR-371a-5p was detected in Caco-2, Caco-2 CR, Caco-2-CRART16 and Caco-2-NC cells by qRT-PCR. U6 was used as an internal control. All experiments were repeated three times, and data are presented as mean ± SD. *P < 0.05, **P < 0.01 by Student’s t test. **e** Dual-luciferase reporter assays in 293T cells. The relative luciferase activity was measured after cotransfection with miR-371a-5p mimics or NC and either the pmiR-RB-Report™-CRART16-WT vector or the empty vector. All experiments were repeated three times, and data are presented as mean ± SD. **P < 0.01 by Student’s t test. **f** The expression of miR-371a-5p was detected by qRT-PCR in Caco-2-CRART16 cells after transfection of miR-371a-5p mimics. U6 was used as an internal control. All experiments were repeated three times, and data are presented as mean ± SD. *P < 0.05, **P < 0.01 by Student’s t test. **g** CCK8 assay was performed to assess the cell viability of Caco-2-CRART16 cells after miR-371a-5p overexpression with graded concentrations of cetuximab (0–200 μg/ml) for 48 h. Each concentration had six replicates, and the experiment was repeated at least three times. Data are presented as mean ± SD. **P < 0.01 by two-way ANOVA

To explore the potential mechanism through which CRART16 confers cetuximab resistance, we first determined the subcellular localization of CRART16. RNA fluorescence in situ hybridization (FISH) showed that CRART16 was located in the cytoplasm and nucleus and was enhanced in Caco-2 CR cells compared with Caco-2 cells (Fig. 4a). Previously published literature indicated that lncRNAs in the cytoplasm can participate in multiple physiological and pathological processes by acting as miRNA sponges. Therefore, RNA sequencing (RNA-seq) analysis was performed on Caco-2 cells overexpressing CRART16. The results showed that 65 miRNAs were upregulated and 39 miRNAs were downregulated in Caco-2-CRART16 cells compared with Caco-2-NC cells (Fig. 4b). Then, we used *TargetScan*, *RNAhybrid* and *MiRanda* to predict whether there are potential binding sites between CRART16 and the downregulated miRNAs. According to the predicted results, CRART16 harbors several binding sites within miR-371a-5p, only three of which are displayed in Fig. 4c. In addition, the expression of miR-371a-5p was measured by qRT-PCR; the expression was lower in Caco-2 CR cells than in Caco-2 cells and was lower in Caco-2-CRART16 cells than in Caco-2-NC cells (Fig. 4d). A dual-luciferase reporter assay was performed to evaluate the interaction between CRART16 and miR-371a-5p (Fig. 4e). Our data showed that the relative luciferase activity was reduced after cotransfection with miR-371a-5p mimics and the CRART16-WT vector, which did not change after cotransfection with NC and the CRART16-WT vector and cotransfection with miR-371a-5p mimics and the empty vector. The results demonstrated that miR-371a-5p was a CRART16-targeting miRNA. In conclusion, CRART16 negatively regulated the expression of miR-371a-5p by directly binding to it, suggesting that CRART16 might act as a sponge for miR-371a-5p. In subsequent experiments, we evaluated the role of miR-371a-5p in cetuximab resistance caused by CRART16 overexpression. The transfection efficiency was analyzed by qRT-PCR 48 h after transfection (Fig. 4f). The CCK8 assay indicated that the overexpression of miR-371a-5p reversed cetuximab resistance in Caco-2-CRART16 cells (Fig. 4g). Taken together, these results suggest that CRART16 contributes to cetuximab resistance by downregulating the expression of miR-371a-5p.

### CRART16 modulates ERBB3 expression in a miR-371a-5p-dependent manner

**Fig. 5:**
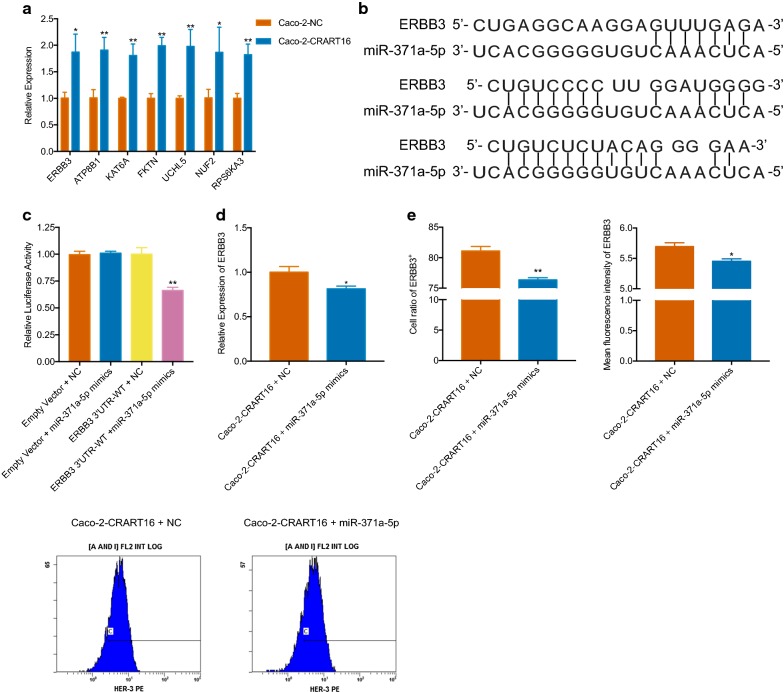
CRART16 modulates ERBB3 expression in a miR-371a-5p-dependent manner. **a** The expression of ERBB3, ATP8B1, KAT6A, FKTN, UCHL5, NUF2 and RPS6KA3 was detected by qRT-PCR after lncRNA CRART16 overexpression. All experiments were repeated three times, and data are presented as mean ± SD. **P < 0.01 by two-way ANOVA. **b** Schematic diagram of the predicted binding sites between ERBB3 and miR-371a-5p. **c** Dual-luciferase reporter assays in 293T cells. The relative luciferase activity was measured after cotransfection with miR-371a-5p mimics or miR-NC and either the pmiR-RB-Report™-ERBB3 3′ UTR-WT vector or the empty vector. All experiments were repeated three times, and data are presented as mean ± SD. **P < 0.01 by Student’s t test. **d** The expression of ERBB3 was detected in Caco-2-CRART16 cells after miR-371a-5p knockdown. All experiments were repeated three times, and data are presented as mean ± SD. *P < 0.05, **P < 0.01 by Student’s t test. **e** The percentage of ERBB3-positive cells and the MFI were determined by GALLIOUS flow cytometry in Caco-2-CRART16 cells after miR-371a-5p knockdown. All experiments were repeated three times, and data are presented as mean ± SD. *P < 0.05, **P < 0.01 by Student’s t test

Based on RNA-seq for mRNA, bioinformatics analysis was performed to predict potential mRNAs downstream of miR-371a-5p. The results indicated that seven mRNAs, which included ERBB3, ATP8B1, KAT6A, FKTN, UCHL5, NUF2 and RPS6KA3, might be targeted by miR-371a-5p. We performed qRT-PCR to confirm the data obtained by RNA-seq (Fig. 5a). Then, we focused on ERBB3, which is the recognized bypass membrane protein of the EGFR signaling pathway. The binding sites between miR-371a-5p and the ERBB3 3′ UTR were predicted by *TargetScan* and *RNAhybrid*, only three of which are displayed in Fig. 5b. MiR-371a-5p mimics, but not NC, repressed the activity of the ERBB3 3′ UTR reporter constructs, as seen in dual-luciferase reporter assays (Fig. 5c). The results demonstrated that ERBB3 was a direct target gene of miR-371a-5p. Subsequently, we carried out a rescue experiment to determine whether CRART16 modulates ERBB3 expression in CRC cells via miR-371a-5p. Caco-2-CRART16 cells were transiently transfected with miR-371a-5p mimics. Two days after transient transfection, ERBB3 mRNA levels were quantified by qRT-PCR. As illustrated in Fig. 5d, miR-371a-5p mimics caused the downregulation of ERBB3 in Caco-2-CRART16 cells. The ERBB3 protein level was assessed by flow cytometry 72 h after transient transfection and also changed concurrently with the mRNA levels (Fig. 5e). Based on these results, we demonstrated that CRART16 upregulated ERBB3 expression in a miR-371a-5p-dependent manner.

### Analysis of differentially expressed genes (DEGs) between Caco-2-CRART16 and Caco-2-NC cells

RNA-seq was further performed to identify the potential signaling pathways underlying the effect of CRART16 in both the Caco-2-NC and Caco-2-CRAR16 cell lines. DEGs were defined as genes with > 2-fold differences and an adjusted P value (padj) < 0.005. Gene Ontology (GO) enrichment analysis and Kyoto Encyclopedia of Genes and Genomes (KEGG) pathway analysis were annotated by ClusterProfiler. GO analysis consists of biological process (BP), molecular function (MF) and cellular component (CC) (Additional file 3: Figure S3a, b). KEGG analysis was performed to identify significantly different pathways between Caco-2-CRART16 and Caco-2-NC cells. As shown in Additional file 3: Figure S3c, we found that the MAPK signaling pathway and ErbB signaling pathway were two differentially enriched pathways. Therefore, we hypothesized that CRART16 might confer cetuximab resistance though the MAPK signaling pathway, which is downstream of EGFR and ERBB3. In addition, DEGs were more strongly related to CRC and EGFR tyrosine kinase inhibitor (TKI) resistance, and these results confirmed the effect of CRART16 on cetuximab resistance. Moreover, the DEGs involved in the four pathways showed a close correlation based on protein–protein interaction (PPI) mapping (Additional file 3: Figure S3d).

## Discussion

In recent years, both the incidence and mortality of CRC have increased in China due to the ‘westernization’ of lifestyle-related factors [20]. Chemotherapy combined with anti-EGFR treatments, such as cetuximab and panitumumab, could significantly improve the outcome of patients with (K)RAS wild-type mCRC [21]. However, mCRC patients develop acquired resistance to cetuximab within 1 year, leading to disease progression. Therefore, the initial purpose of this study was to identify whether lncRNAs confer cetuximab resistance. In this study, we focused on the novel lncRNA CRART16, which was identified by an RNA microarray and is upregulated during acquired cetuximab resistance in a CRC cell line. Since CRART16 expression was observed in the cytoplasm, we hypothesized that CRART16 exerts its effects by acting as a miRNA sponge. After overexpressing CRART16 in the Caco-2 cell line, RNA-seq analysis was performed. Combined with bioinformatics analysis, CRART16 caused cetuximab resistance by binding to miR-371a-5p, resulting in an increase in ERBB3 expression, which was experimentally verified. Additionally, CRART16 contributed to the acquisition of stemness properties in CRC cells.

In recent years, numerous lncRNAs, originally named long RNAs (lRNAs) [22], have been identified by researchers and have become research hotspots in the field of medicine, especially in cancer initiation, promotion, and progression. For example, both the lncRNA HNF1A-antisense 1 (HNF1A-AS1) and the lncRNA nuclear-enriched abundant transcript 1 (NEAT1) are upregulated in colon cancer tissues, promote the proliferation and invasion of CRC cells and function as ceRNAs to modulate miRNA-34a expression, subsequently causing the repression of the miR-34a/SIRT1 axis [23, 24]. The lncRNA plasmacytoma variant translocation 1 (PVT1)-214 acts as an oncogene that can facilitate proliferation, migration, and invasion in CRC cells by reducing Lin28 protein degradation and enhancing its stability and by increasing Lin28 at the posttranscriptional level by binding to miR-128 [25]. However, there has been little research on the role of lncRNAs in cetuximab resistance. The downregulation of lncRNA POU class 5 homeobox 1 pseudogene 4 (POU5F1P4) expression led to cetuximab resistance in CRC cells [26]. Conversely, the knockdown of LINC00973, which is upregulated in cetuximab-resistant cells, ameliorated the resistance of CRC cells to cetuximab [27]. In this study, we draw attention to the role of CRART16 in acquired cetuximab resistance. The overexpression of CRART16 has been shown to decrease the sensitivity of CRC cells to cetuximab by various experiments.

Noncoding RNAs that are 21–25 nt in length were first recognized in 1993 [28] and were named miRNAs in 2001 [29–31]. MiRNAs can act as oncogenes and tumor suppressor genes, leading to the degradation of downstream mRNAs by binding to complementary sequences in the 3′ UTR of mRNAs [32]. In addition, miRNAs can participate in anticancer therapy resistance, thus affecting patient prognosis. MiR-100 and miR-125b were upregulated in cetuximab-resistant cells, and this result is consistent with our RNA-seq results (Fig. 4b); miR-100 and miR-125b cooperativity induced cetuximab resistance by elevating the activity of the Wnt signaling pathway [33]. Likewise, the decreased expression of miR-199a-5p and miR-375 can increase sensitivity to cetuximab by enhancing PHLPP1 expression [34]. According to bioinformatics analysis and experimental validation by dual-luciferase reporter assays, our findings suggest that CRART16 overexpression increased ERBB3 expression by binding to miR-371a-5p. Several previous studies have shown that miR-371a-5p contributes to the development and progression of different cancers, such as hepatocellular carcinoma (HCC) [35] and pancreatic carcinoma [36]. In this study, a rescue assay indicated that CRART16 overexpression-induced cetuximab resistance was partially counteracted by miR-371a-5p mimics, which suggested that CRART16 might also act through other mechanisms.

EGFR/Her1, ERBB2/Her2, ERBB3/Her3 and ERBB4/Her4 are members of the v-erb-b2 erythroblastic leukemia viral oncogene (ErbB)/human epidermal receptor (HER) family of transmembrane receptor tyrosine kinases (RTKs) [37]. Moreover, MET is an RTK for hepatocyte growth factor (HGF), which is involved in signaling crosstalk with EGFR [38, 39]. Previous studies demonstrated that ERBB3 and MET are a part of the typical bypass mechanism in ERBB family TKI therapy, which could activate the downstream pathway [39–42]. In addition, MET amplification led to the continued activation of the downstream PI3K pathway by maintaining the phosphorylation of ERBB3. In our study, flow cytometry analysis showed that EGFR was rarely expressed in Caco-2 CR cells due to continued stimulation with cetuximab. On the other hand, the MFIs of ERBB3 and MET showed a compensatory increase in Caco-2 CR cells. Compared with Caco-2-NC cells, Caco-2-CRART16 cells showed a decreased MFI of EGFR and an increased MFI of MET. Importantly, both the MFI of ERBB3 and the ERBB3^+^ cell ratio were increased in Caco-2-CRART16 cells. In addition, miR-371a-5p overexpression in Caco-2-CRART16 cells decreased both the MFI of ERBB3 and the cell ratio of ERBB3^+^, and these results are in line with the assumption that CRART16 positively regulated ERBB3 by binding to miR-371a-5p. However, the regulatory mechanism of CRART16 for EGFR and MET is worthy of further research.

The presence of CSC subpopulations has been shown to be associated with tumor initiation, drug and radiation resistance, invasive growth, metastasis, and tumor relapse [43]. CD44 is a transmembrane glycoprotein that has been recognized as a CSC marker in a variety of cancers [44]. In CRC, CD44 overexpression is related to poor differentiation, lymph node metastasis and distant metastasis [45]. CD133, also known as prominin-1, is negatively correlated with OS in CRC patients [46]. Moreover, the double-positivity of CD133/CD44 is a reliable biomarker for the identification and isolation of CSCs in CRC cells [46]. Previous studies demonstrated that CD133^high^/CD44^high^-expressing CRC cells could have an increased resistance to radiation [47]. In addition, the expression of CD44 and CD133 was increased in CRC cells with acquired resistance to an anti-EGFR monoclonal antibody (mAb) and TKI therapies [48]. In agreement with these data, our findings suggested that Caco-2 CR cells contained a higher percentage of CD44^+^/CD133^+^ cells than Caco-2 cells, and this result demonstrates that CSCs are involved in not only primary resistance but also in acquired resistance. Additionally, CRART16 overexpression also promoted the acquisition of stemness properties.

## Conclusions

**Fig. 6:**
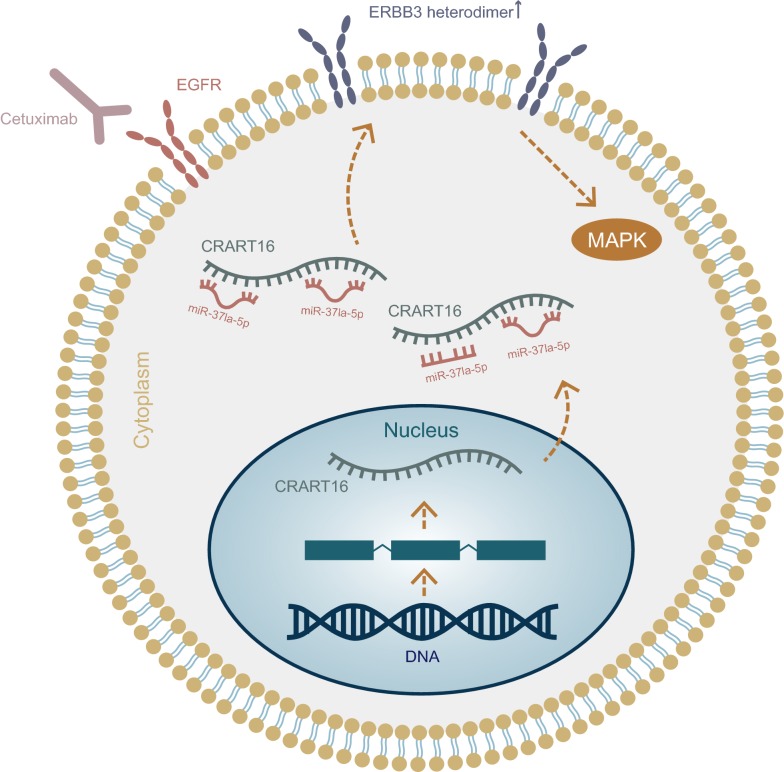
Schematic diagram of the mechanism of CRART16 in cetuximab resistance

Our results illustrate that CRART16 is upregulated in acquired cetuximab-resistant CRC cells. CRART16 confers cetuximab resistance in CRC cells by acting as a miR-371a-5p sponge and by subsequently increasing the expression of ERBB3 (Fig. 6). Additionally, CRART16 overexpression can promote CSC transformation. Taken together, CRART16 could be used as a marker to predict sensitivity to cetuximab and as a potential therapeutic target for cetuximab resistance.

## Supplementary information

**Additional file 1: Figure S1.** Phenotypic characteristics of Caco-2 and Caco-2 CR cells. **a** Flow cytometry was performed in Caco-2 and Caco-2 CR cells with cetuximab treatment (100 μg/ml and 200 μg/ml) for 48 h. APC Annexin V^−^/7-AAD^−^ denotes live cells; APC Annexin V^+^/7-AAD^−^ denotes early apoptotic cells; APC Annexin V^−^/7-AAD^+^ denotes necrotic cells; and APC Annexin V^+^/7-AAD^+^ denotes late apoptotic cells. **b** The cell cycle was assessed by flow cytometry in Caco-2 and Caco-2 CR cells after 48 h of treatment with cetuximab (200 μg/ml). **c** The percentage of EGFR-, ERBB3-, and c-MET-positive cells and the MFI were determined by GALLIOUS flow cytometry in Caco-2 and Caco-2 CR cells. **d** Flow cytometry analysis showed the expression of stemness biomarkers of CRC cells, CD44 and CD133, in Caco-2 and Caco-2 CR cells.

**Additional file 2: Figure S2.** CRART16 promotes cetuximab resistance and contributes to the acquisition of stemness properties of CRC cells. **a** Flow cytometry was performed in Caco-2-CRART16 and Caco-2-NC cells with cetuximab treatment (100 μg/ml and 200 μg/ml) for 48 h. APC Annexin V^−^/7-AAD^−^ denotes live cells; APC Annexin V^+^/7-AAD^−^ denotes early apoptotic cells; APC Annexin V^−^/7-AAD^+^ denotes necrotic cells; and APC Annexin V^+^/7-AAD^+^ denotes late apoptotic cells. **b** The cell cycle was assessed by flow cytometry in Caco-2-CRART16 and Caco-2-NC cells after 48 h of treatment with cetuximab (200 μg/ml). **c** The percentage of EGFR-, ERBB3-, and c-MET-positive cells and the MFI were determined by a GALLIOUS flow cytometer in Caco-2-CRART16 and Caco-2-NC cells. **d** Flow cytometry analysis showed the expression of stemness biomarkers in CRC cells, CD44 and CD133, in Caco-2-CRART16 and Caco-2-NC cells.

**Additional file 3: Figure S3.** Gene-set enrichment analysis between Caco-2-CRART16 and Caco-2-NC cells. **a**, **b** GO analysis. **c** KEGG analysis. **d** PPI mapping.

## Data Availability

The datasets generated during and/or analyzed during the current study are available from the corresponding author on reasonable request.

## Abbreviations

BP: Biological process
Caco-2 CR: Caco-2 cetuximab-resistant cells
CC: Cellular component
CCK-8: Cell Counting Kit-8
cDNA: Complementary DNA
ceRNA: Competing endogenous RNA
CO_2_: Carbon dioxide
CRART16: Cetuximab resistance-associated RNA transcript 16
CRC: Colorectal cancer
cRNA: Complementary RNA
CSC: Cancer stem cell
DAB: Diaminobenzene
DMEM: Dulbecco’s modified Eagle’s medium–high glucose
dsDNA: Double-stranded cDNA
EGFR: Epidermal growth factor receptor
ErbB: v-erb-b2 erythroblastic leukemia viral oncogene
FBS: Fetal bovine serum
FISH: Fluorescence in situ hybridization
GO: Gene Ontology
HCC: Hepatocellular carcinoma
HER: Human epidermal receptor
HGF: Hepatocyte growth factor
HGNC: HUGO Gene Nomenclature Committee
HNF1A-AS1: lncRNA HNF1A-antisense 1
IgG1: Immunoglobulin G1
KEGG: Kyoto Encyclopedia of Genes and Genomes
lncRNA: Long noncoding RNA
lRNA: Long RNA
mAb: Monoclonal antibody
mCRC: Metastatic colorectal cancer
MF: Molecular function
MFI: Mean fluorescence intensity
miRNA: microRNA
miR-NC: Negative control RNA
NEAT1: lncRNA nuclear-enriched abundant transcript 1
nt: Nucleotide
OS: Overall survival
padj: Adjusted P value
POU5F1P4: lncRNA POU class 5 homeobox 1 pseudogene 4
PPI: Protein–protein interaction
PVT1: lncRNA plasmacytoma variant translocation 1
qRT-PCR: Quantitative real-time PCR
RTK: Transmembrane receptor tyrosine kinase
snRNA: Small nuclear RNA
TKI: Tyrosine kinase inhibitor
3′ UTR: 3′ untranslated region
7-AAD: 7-Amino-actinomycin D

## References

[1] Bray F, Ferlay J, Soerjomataram I, Siegel RL, Torre LA, Jemal A (2018). Global cancer statistics 2018: GLOBOCAN estimates of incidence and mortality worldwide for 36 cancers in 185 countries. CA Cancer J Clin.

[2] Cunningham D, Atkin W, Lenz HJ, Lynch HT, Minsky B, Nordlinger B (2010). Colorectal cancer. Lancet.

[3] Stintzing S, Modest DP, Rossius L, Lerch MM, von Weikersthal LF, Decker T (2016). FOLFIRI plus cetuximab versus FOLFIRI plus bevacizumab for metastatic colorectal cancer (FIRE-3): a post hoc analysis of tumour dynamics in the final RAS wild-type subgroup of this randomised open-label phase 3 trial. Lancet Oncol..

[4] Venook AP, Niedzwiecki D, Lenz HJ, Innocenti F, Fruth B, Meyerhardt JA (2017). Effect of first-line chemotherapy combined with cetuximab or bevacizumab on overall survival in patients with KRAS wild-type advanced or metastatic colorectal cancer: a randomized clinical trial. JAMA.

[5] Ciardiello F, Tortora G (2008). EGFR antagonists in cancer treatment. N Engl J Med.

[6] Oda K, Matsuoka Y, Funahashi A, Kitano H (2005). A comprehensive pathway map of epidermal growth factor receptor signaling. Mol Syst Biol..

[7] De Roock W, Claes B, Bernasconi D, De Schutter J, Biesmans B, Fountzilas G (2010). Effects of KRAS, BRAF, NRAS, and PIK3CA mutations on the efficacy of cetuximab plus chemotherapy in chemotherapy-refractory metastatic colorectal cancer: a retrospective consortium analysis. Lancet Oncol..

[8] Zhao B, Wang L, Qiu H, Zhang M, Sun L, Peng P (2017). Mechanisms of resistance to anti-EGFR therapy in colorectal cancer. Oncotarget..

[9] Van Emburgh BO, Sartore-Bianchi A, Di Nicolantonio F, Siena S, Bardelli A (2014). Acquired resistance to EGFR-targeted therapies in colorectal cancer. Mol Oncol..

[10] Derrien T, Johnson R, Bussotti G, Tanzer A, Djebali S, Tilgner H (2012). The GENCODE v7 catalog of human long noncoding RNAs: analysis of their gene structure, evolution, and expression. Genome Res.

[11] Fatica A, Bozzoni I (2014). Long non-coding RNAs: new players in cell differentiation and development. Nat Rev Genet.

[12] Huarte M (2015). The emerging role of lncRNAs in cancer. Nat Med.

[13] Xiong XD, Ren X, Cai MY, Yang JW, Liu X, Yang JM (2016). Long non-coding RNAs: an emerging powerhouse in the battle between life and death of tumor cells. Drug Resist Updates.

[14] Wang KC, Chang HY (2011). Molecular mechanisms of long noncoding RNAs. Mol Cell.

[15] Chen LL (2016). Linking Long Noncoding RNA Localization and Function. Trends Biochem Sci.

[16] Kartha RV, Subramanian S (2014). Competing endogenous RNAs (ceRNAs): new entrants to the intricacies of gene regulation. Front Genet..

[17] Han P, Li JW, Zhang BM, Lv JC, Li YM, Gu XY (2017). The lncRNA CRNDE promotes colorectal cancer cell proliferation and chemoresistance via miR-181a-5p-mediated regulation of Wnt/beta-catenin signaling. Mol Cancer..

[18] Xu M, Chen X, Lin K, Zeng K, Liu X, Xu X (2019). lncRNA SNHG6 regulates EZH2 expression by sponging miR-26a/b and miR-214 in colorectal cancer. J Hematol Oncol..

[19] Wright MW (2014). A short guide to long non-coding RNA gene nomenclature. Hum Genomics..

[20] Arnold M, Sierra MS, Laversanne M, Soerjomataram I, Jemal A, Bray F (2017). Global patterns and trends in colorectal cancer incidence and mortality. Gut.

[21] van Helden EJ, Menke-van der Houven van Oordt CW, Heymans MW, Ket JCF, van den Oord R, Verheul HMW (2017). Optimal use of anti-EGFR monoclonal antibodies for patients with advanced colorectal cancer: a meta-analysis. Cancer Metastasis Rev..

[22] Kapranov P, Cheng J, Dike S, Nix DA, Duttagupta R, Willingham AT (2007). RNA maps reveal new RNA classes and a possible function for pervasive transcription. Science.

[23] Luo Y, Chen JJ, Lv Q, Qin J, Huang YZ, Yu MH (2019). Long non-coding RNA NEAT1 promotes colorectal cancer progression by competitively binding miR-34a with SIRT1 and enhancing the Wnt/beta-catenin signaling pathway. Cancer Lett.

[24] Fang C, Qiu S, Sun F, Li W, Wang Z, Yue B (2017). Long non-coding RNA HNF1A-AS1 mediated repression of miR-34a/SIRT1/p53 feedback loop promotes the metastatic progression of colon cancer by functioning as a competing endogenous RNA. Cancer Lett.

[25] He F, Song Z, Chen H, Chen Z, Yang P, Li W (2019). Long noncoding RNA PVT1-214 promotes proliferation and invasion of colorectal cancer by stabilizing Lin28 and interacting with miR-128. Oncogene.

[26] Peng K, Liu R, Yu Y, Liang L, Yu S, Xu X (2018). Identification and validation of cetuximab resistance associated long noncoding RNA biomarkers in metastatic colorectal cancer. Biomed Pharmacother.

[27] Jing C, Ma R, Cao H, Wang Z, Liu S, Chen D (2019). Long noncoding RNA and mRNA profiling in cetuximab-resistant colorectal cancer cells by RNA sequencing analysis. Cancer Med.

[28] Lee RC, Feinbaum RL, Ambros V (1993). The *C elegans* heterochronic gene lin-4 encodes small RNAs with antisense complementarity to lin-14. Cell..

[29] Lagos-Quintana M, Rauhut R, Lendeckel W, Tuschl T (2001). Identification of novel genes coding for small expressed RNAs. Science.

[30] Lau NC, Lim LP, Weinstein EG, Bartel DP (2001). An abundant class of tiny RNAs with probable regulatory roles in Caenorhabditis elegans. Science.

[31] Lee RC, Ambros V (2001). An extensive class of small RNAs in *Caenorhabditis elegans*. Science.

[32] Lin S, Gregory RI (2015). MicroRNA biogenesis pathways in cancer. Nat Rev Cancer.

[33] Lu Y, Zhao X, Liu Q, Li C, Graves-Deal R, Cao Z (2017). lncRNA MIR100HG-derived miR-100 and miR-125b mediate cetuximab resistance via Wnt/beta-catenin signaling. Nat Med.

[34] Mussnich P, Rosa R, Bianco R, Fusco A, D’Angelo D (2015). MiR-199a-5p and miR-375 affect colon cancer cell sensitivity to cetuximab by targeting PHLPP1. Expert Opin Ther Targets..

[35] Bai PS, Hou P, Kong Y (2018). Hepatitis B virus promotes proliferation and metastasis in male Chinese hepatocellular carcinoma patients through the LEF-1/miR-371a-5p/SRCIN1/pleiotrophin/Slug pathway. Exp Cell Res.

[36] Yue L, Guo J (2019). LncRNA TUSC7 suppresses pancreatic carcinoma progression by modulating miR-371a-5p expression. J Cell Physiol.

[37] Yarden Y, Sliwkowski MX (2001). Untangling the ErbB signalling network. Nat Rev Mol Cell Biol.

[38] Gherardi E, Birchmeier W, Birchmeier C, Woude GV (2012). Targeting MET in cancer: rationale and progress. Nat Rev Cancer.

[39] Troiani T, Martinelli E, Napolitano S, Vitagliano D, Ciuffreda LP, Costantino S (2013). Increased TGF-alpha as a mechanism of acquired resistance to the anti-EGFR inhibitor cetuximab through EGFR-MET interaction and activation of MET signaling in colon cancer cells. Clin Cancer Res.

[40] Sergina NV, Rausch M, Wang D, Blair J, Hann B, Shokat KM (2007). Escape from HER-family tyrosine kinase inhibitor therapy by the kinase-inactive HER3. Nature.

[41] Liska D, Chen CT, Bachleitner-Hofmann T, Christensen JG, Weiser MR (2011). HGF rescues colorectal cancer cells from EGFR inhibition via MET activation. Clin Cancer Res.

[42] Bosch-Vilaro A, Jacobs B, Pomella V, Asbagh LA, Kirkland R, Michel J (2017). Feedback activation of HER3 attenuates response to EGFR inhibitors in colon cancer cells. Oncotarget..

[43] Taniguchi H, Moriya C, Igarashi H, Saitoh A, Yamamoto H, Adachi Y (2016). Cancer stem cells in human gastrointestinal cancer. Cancer Sci.

[44] Morath I, Hartmann TN, Orian-Rousseau V (2016). CD44: more than a mere stem cell marker. Int J Biochem Cell Biol.

[45] Wang Z, Tang Y, Xie L, Huang A, Xue C, Gu Z (2019). The prognostic and clinical value of CD44 in colorectal cancer: a meta-analysis. Front Oncol..

[46] Abbasian M, Mousavi E, Arab-Bafrani Z, Sahebkar A (2019). The most reliable surface marker for the identification of colorectal cancer stem-like cells: a systematic review and meta-analysis. J Cell Physiol.

[47] Sahlberg SH, Spiegelberg D, Glimelius B, Stenerlow B, Nestor M (2014). Evaluation of cancer stem cell markers CD133, CD44, CD24: association with AKT isoforms and radiation resistance in colon cancer cells. PLoS ONE.

[48] Khelwatty SA, Essapen S, Bagwan I, Green M, Seddon AM, Modjtahedi H (2019). Co-expression and prognostic significance of putative CSC markers CD44, CD133, wild-type EGFR and EGFRvIII in metastatic colorectal cancer. Oncotarget..

