# Supplementary figures and images for: The novel long noncoding RNA CRART16 confers cetuximab resistance in colorectal cancer cells by enhancing ERBB3 expression via miR-371a-5p
Source: Cancer Cell Int. 2020 Mar 4;20:68. doi: 10.1186/s12935-020-1155-9 (PMC7057486; doi:10.1186/s12935-020-1155-9)

**a**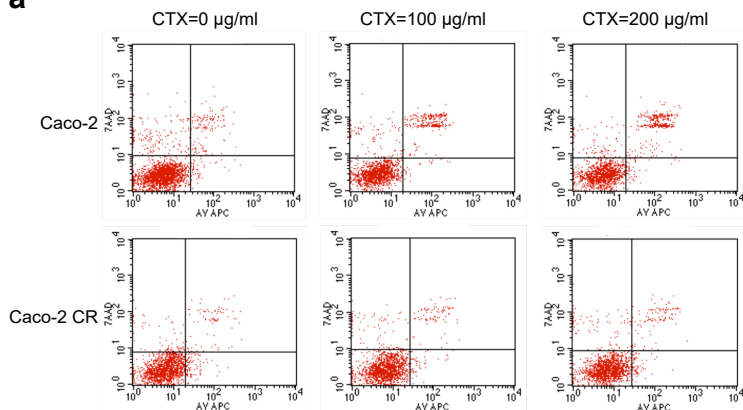**b**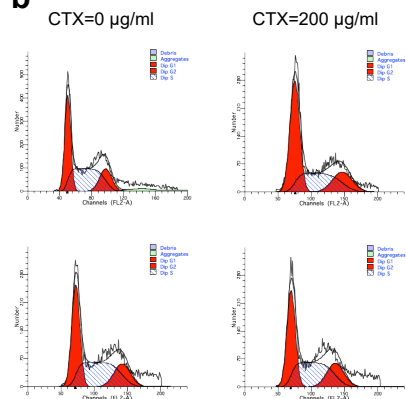**c**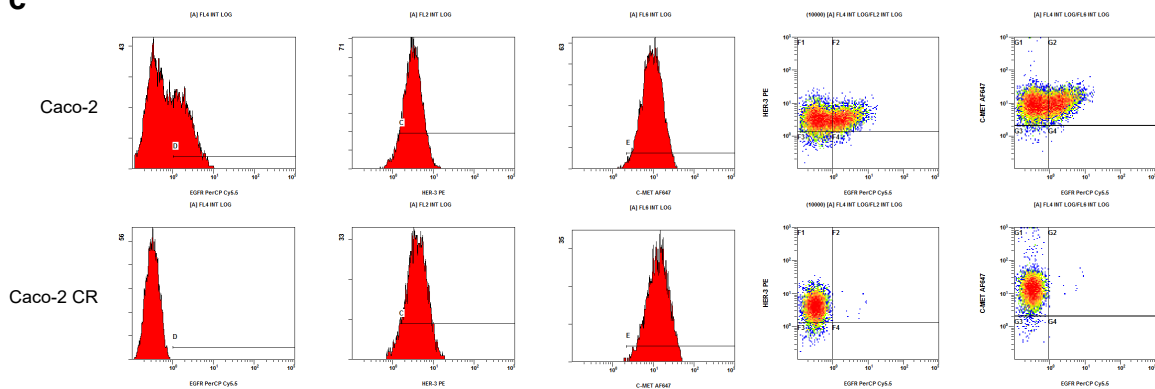**d**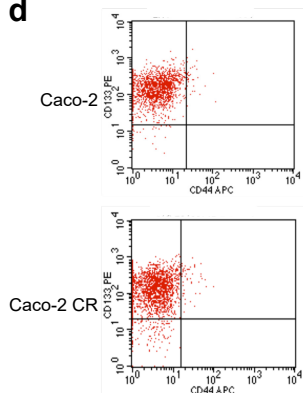

Supplement: Supplementary file 1 — Additional file 1: Figure S1. Phenotypic characteristics of Caco-2 and Caco-2 CR cells. a Flow cytometry was performed in Caco-2 and Caco-2 CR cells with cetuximab treatment (100 μg/ml and 200 μg/ml) for 48 h. APC Annexin V−/7-AAD− denotes live cells; APC Annexin V+/7-AAD− denotes early apoptotic cells; APC Annexin V−/7-AAD+ denotes necrotic cells; and APC Annexin V+/7-AAD+ denotes late apoptotic cells. b The cell cycle was assessed by flow cytometry in Caco-2 and Caco-2 CR cells after 48 h of treatment with cetuximab (200 μg/ml). c The percentage of EGFR-, ERBB3-, and c-MET-positive cells and the MFI were determined by GALLIOUS flow cytometry in Caco-2 and Caco-2 CR cells. d Flow cytometry analysis showed the expression of stemness biomarkers of CRC cells, CD44 and CD133, in Caco-2 and Caco-2 CR cells. [file 12935_2020_1155_MOESM1_ESM.pdf]

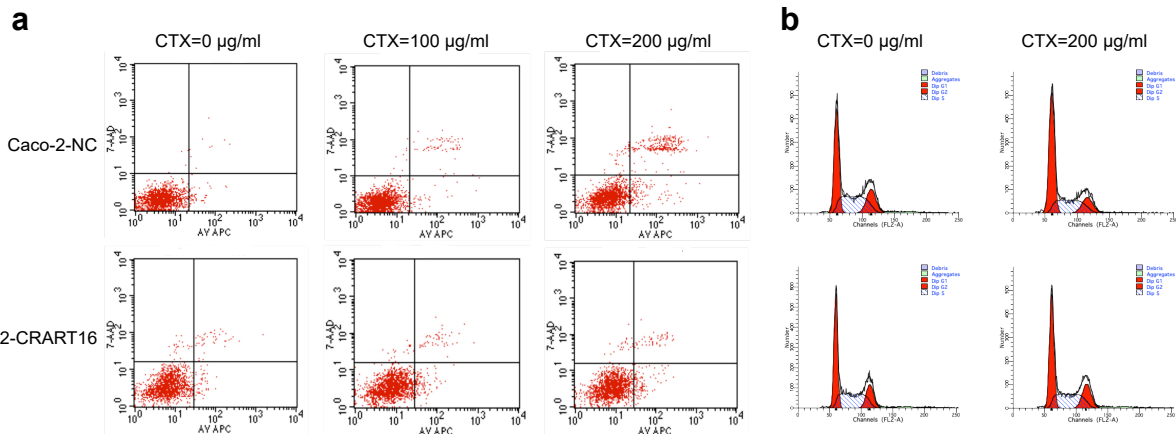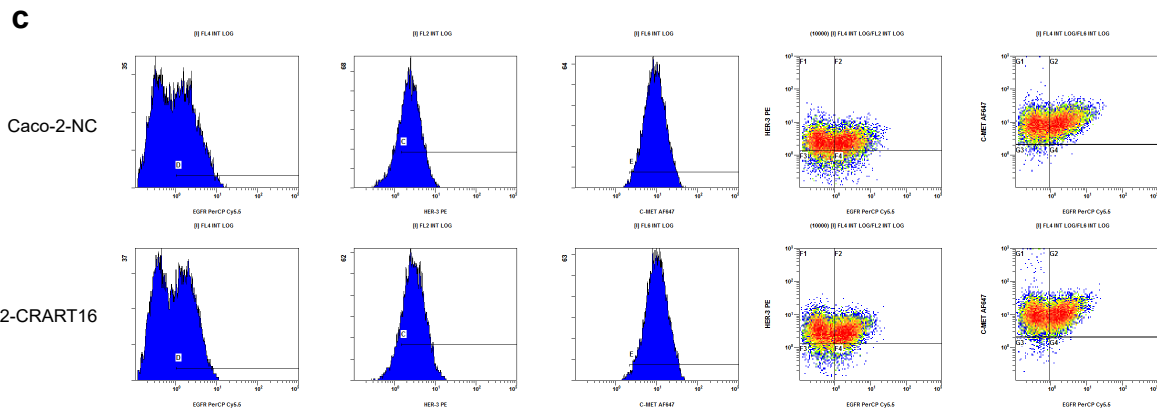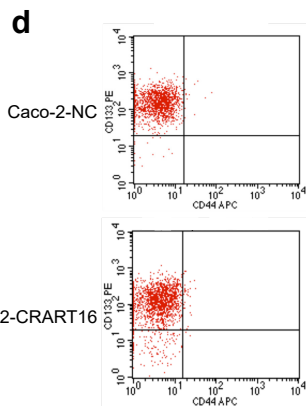

Supplement: Supplementary file 2 — Additional file 2: Figure S2. CRART16 promotes cetuximab resistance and contributes to the acquisition of stemness properties of CRC cells. a Flow cytometry was performed in Caco-2-CRART16 and Caco-2-NC cells with cetuximab treatment (100 μg/ml and 200 μg/ml) for 48 h. APC Annexin V−/7-AAD− denotes live cells; APC Annexin V+/7-AAD− denotes early apoptotic cells; APC Annexin V−/7-AAD+ denotes necrotic cells; and APC Annexin V+/7-AAD+ denotes late apoptotic cells. b The cell cycle was assessed by flow cytometry in Caco-2-CRART16 and Caco-2-NC cells after 48 h of treatment with cetuximab (200 μg/ml). c The percentage of EGFR-, ERBB3-, and c-MET-positive cells and the MFI were determined by a GALLIOUS flow cytometer in Caco-2-CRART16 and Caco-2-NC cells. d Flow cytometry analysis showed the expression of stemness biomarkers in CRC cells, CD44 and CD133, in Caco-2-CRART16 and Caco-2-NC cells. [file 12935_2020_1155_MOESM2_ESM.pdf]
