# Supplementary material for: The novel long noncoding RNA CRART16 confers cetuximab resistance in colorectal cancer cells by enhancing ERBB3 expression via miR-371a-5p
Source: Cancer Cell Int. 2020 Mar 4;20:68. doi: 10.1186/s12935-020-1155-9 (PMC7057486; doi:10.1186/s12935-020-1155-9)
Supplement: Supplementary file 3 — Additional file 3: Figure S3. Gene-set enrichment analysis between Caco-2-CRART16 and Caco-2-NC cells. a, b GO analysis. c KEGG analysis. d PPI mapping. [file 12935_2020_1155_MOESM3_ESM.pdf]

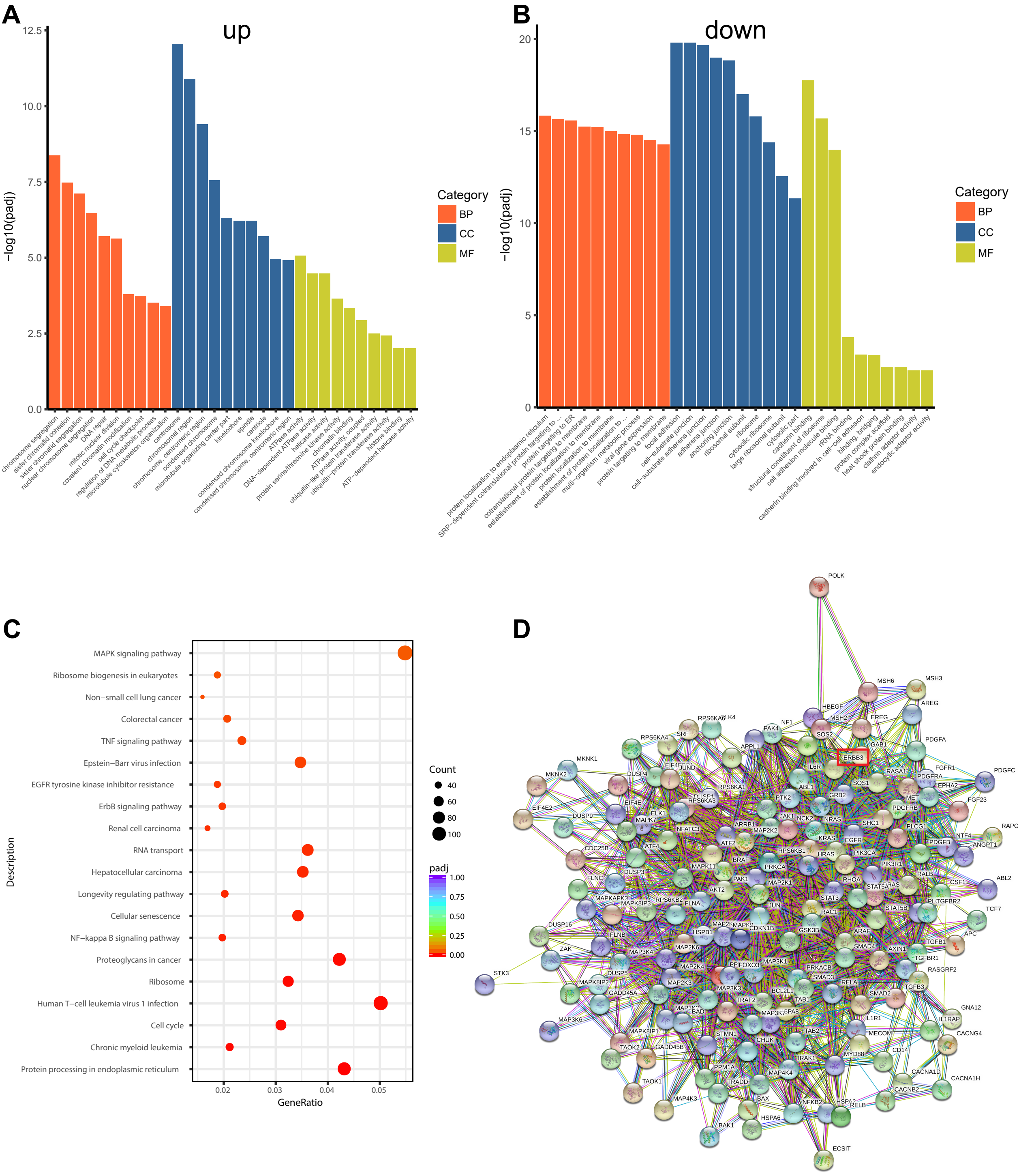
A
up
-log10(padj)
12.5
10.0
7.5
5.0
2.5
0.0
Category
BP
CC
MF
chromosome segregation
sister chromatid cohesion
sister chromatid segregation
nuclear chromosome segregation
DNA repair
mitotic nuclear division
covalent chromatin modification
cell cycle checkpoint
regulation of DNA metabolic process
microtubule cytoskeleton organization
centrosome
chromosomal region
chromosome, centromeric region
condensed chromosome
microtubule organizing center part
kinetochore
spindle
centriole
condensed chromosome kinetochore
condensed chromosome, centromeric region
ATPase activity
DNA−dependent ATPase activity
helicase activity
protein serine/threonine kinase activity
chromatin binding
ATPase activity, coupled
ubiquitin−like protein transferase activity
ubiquitin−protein transferase activity
histone binding
ATP−dependent helicase activity
B
down
-log10(padj)
20
15
10
5
0
Category
BP
CC
MF
protein localization to endoplasmic reticulum
SRP−dependent cotranslational protein targeting to ...
protein targeting to ER
cotranslational protein targeting to membrane
establishment of protein localization to membrane
protein localization to membrane
establishment of protein localization to ...
multi−organism metabolic process
viral gene expression
protein targeting to membrane
focal adhesion
cell−substrate adherens junction
cell−substrate junction
adherens junction
anchoring junction
ribosomal subunit
ribosome
cytosolic ribosome
large ribosomal subunit
cytosolic part
cadherin binding
structural constituent of ribosome
cell adhesion molecule binding
cadherin binding involved in cell−cell adhesion
rRNA binding
binding, bridging
protein complex scaffold
heat shock protein binding
clathrin adaptor activity
endocytic adaptor activity
C
Description
MAPK signaling pathway
Ribosome biogenesis in eukaryotes
Non−small cell lung cancer
Colorectal cancer
TNF signaling pathway
Epstein−Barr virus infection
EGFR tyrosine kinase inhibitor resistance
ErbB signaling pathway
Renal cell carcinoma
RNA transport
Hepatocellular carcinoma
Longevity regulating pathway
Cellular senescence
NF−kappa B signaling pathway
Proteoglycans in cancer
Ribosome
Human T−cell leukemia virus 1 infection
Cell cycle
Chronic myeloid leukemia
Protein processing in endoplasmic reticulum
0.02
0.03
0.04
0.05
GeneRatio
Count
40
60
80
100
padj
1.00
0.75
0.50
0.25
0.00
D
POLK
MSH6
MSH3
AREG
HBEGF
MSH2
EREG
NF1
PAK4
SOS2
GAB1
PDGFA
ERBB3
RPS6KA4
SRF
APPL1
MKNK1
MKNK2
DUSP4
IL6R
FGFR1
RASA1
PDGFRA
PDGFC
EPHA2
SOS1
ABL1
RPS6KA1
GRB2
MET
FGF23
EIF4E2
EIF4E
ELK1
PTK2
PDGFRB
DUSP9
MAPK7
JAK1
NCK2
NRAS
SHC1
PLCG1
RAPG
NFATC3
ARRB1
MAP2K2
KRAS
EGFR
NTF4
ANGPT1
PDGFB
CDC25B
ATF4
ATF2
RPS6KB1
HRAS
PIK3CA
BRAF
PRKCA
PIK3R1
RALB
DUSP3
MAPK11
ABL2
FLNC
PAK1
MAP2K1
RHOA
STAT5A
CSF1
AKT2
STAT3
TGFBR2
MAPKAPK3
MAPK8IP3
RPS6KB2
FLNA
JUN
STAT5B
TCF7
RAC1
DUSP16
FLNB
CDKN1B
GSK3B
ARAF
APC
HSPB1
SMAD4
AXIN1
TGFB1
ZAK
MAP2K6
MAP3K4
TGFBR1
MAP2K4
FOXO3
MAP3K1
PRKACB
RASGRF2
STK3
DUSP5
SMAD3
MAP2K3
RELA
MAPK8IP2
TGFB3
GADD45A
MAP3K3
BCL2L1
SMAD2
TRAF2
TAB1
BAD
MAP3K7
GNA12
MAP3K6
IL1R1
IL1RAP
MAPK8IP1
STMN1
TAB2
MECOM
CACNG4
TAOK2
GADD45B
CHUK
CD14
IRAK1
MYD88
PPM1A
MAP4K4
CACNA1D
CACNA1H
TAOK1
TRADD
CACNB2
MAP4K3
BAX
NFKB2
HSPA2
RELB
HSPA6
BAK1
ECSIT
